# Low expression of CDHR1 is an independent unfavorable prognostic factor in glioma

**DOI:** 10.7150/jca.59948

**Published:** 2021-06-26

**Authors:** Haiwei Wang, Xinrui Wang, Liangpu Xu, Yingying Lin, Ji Zhang, Hua Cao

**Affiliations:** 1 Medical Research Center, Fujian Maternity and Child Health Hospital, Affiliated Hospital of Fujian Medical University, Fuzhou, Fujian, China.; 2 Key Laboratory of Technical Evaluation of Fertility Regulation for Non-human Primate, National Health and Family Planning Commission, Fuzhou, Fujian, China.; 3 Department of neurosurgery, Renji Hospital, Shanghai Jiao Tong University School of Medicine, Shanghai, China.; 4 State Key Laboratory for Medical Genomics, Shanghai Institute of Hematology, Rui-Jin Hospital Affiliated to School of Medicine, Shanghai Jiao Tong University, Shanghai, China.

**Keywords:** CDHR1, Lower grade glioma, Glioblastoma, TCGA, GEO datasets

## Abstract

**Background:** Analysis of the differentially expressed genes between lower grade glioma (LGG) and glioblastoma (GBM) will identify genes involved in a more aggressive phenotype of glioma.

**Methods:** Differentially expressed genes between GBM and LGG were identified using published datasets. Kaplan-Meier estimator was used to determine the overall survival of different groups of glioma patients. The biological functions of CDHR1 in glioma were tested using CCK-8 and trans-well assays.

**Results:** CCDC109B, CD58, CLIC1, EFEMP2, EMP3, LAMC1, LGALS1, PDLIM1 and TNFRSF1A were over-expressed, while, CDHR1 was down-regulated in GBM in The Cancer Genome Atlas (TCGA), Chinese Glioma Genome Atlas (CGGA), GSE4412 and GSE43378 datasets. Compared with normal brain tissues, CDHR1 was down-regulated in glioma tissues. And low expression of CDHR1 was an unfavorable prognostic factor in glioma. Moreover, CDHR1 was lowly expressed in mesenchymal GBM subtype and lower expression of CDHR1 was associated with the worse clinical prognosis of GBM. Furthermore, CDHR1 was down-regulated in astrocytoma LGG subtype and low expression of CDHR1 was a bad prognosis of LGG. CDHR1 expression levels were also associated with IDH mutation. IDH mutant LGG or GBM patients were with higher CDHR1 expression. High expression of CDHR1 was a favorable prognosis in IDH mutant or IDH wild type LGG patients. CHDR1 expression was associated with MGMT methylation and CDHR1 was down-regulated in chemotherapy un-responsive LGG patients. CDHR1 was an independent prognostic factor and negatively associated with EMP3 expression. Glioma patients with low CDHR1 and high EMP3 expression had worse clinical outcomes. At last, we showed that over-expression of CDHR1 could inhibit glioma cell growth and invasion.

**Conclusion:** Low expression of CDHR1 was an independent unfavorable prognostic factor in glioma.

## Introduction

Glioma is originated from the central nervous system, representing the most common and aggressive type of brain tumor 1, 2. Although with the deep understanding of the molecular alterations of glioma, therapeutic options for glioma patients are still limited to surgery, chemotherapy and radiation therapy 3, 4. Moreover, most of glioma patients become therapeutic resistant and recurrent during the treatment 5, 6. So, it is important to search novel molecular targets and prognostic makers to predict the therapeutic responses and clinical outcomes of glioma.

Glioma could be divided into lower grade glioma (LGG) and glioblastoma (GBM) based on the histological grades 7. LGG is grade II-III glioma and GBM is grade IV glioma. The overall survival of patients with LGG is about 5-10 years 8. On the contrary, the median survival of patients with GBM is only 12 to 15 months 9, 10. Although, most primary GBMs are developed *de novo*, without effective treatments, some LGG patients would eventually progress to more malignant GBM 11, 12. The molecular characteristics of LGG and GBM are extensively studied by The Cancer Genome Atlas (TCGA) 13, 14 and Chinese Glioma Genome Atlas (CGGA) 15, 16 groups, respectively. However, the differentially expressed genes between GBM and LGG are still not very clear. We thought that identification of the transcriptional signatures of different grades of glioma may provide new prognostic makers.

Cadherin-related family member 1 (CDHR1) is a photoreceptor-specific cadherin, belonging to the cadherin super-family 17. Mutations of CDHR1 are detected in retinal dystrophies disease 18. However, the functions of CDHR1 in tumor development are barely studied. Moreover, the expression and prognostic significance of CDHR1 in glioma are never reported. Here, using TCGA, CGGA and Gene Expression Omnibus (GEO) datasets, we studied the differentially expressed genes between GBM and LGG, and our data suggested that low expression of CDHR1 was an independent unfavorable prognostic factor in glioma.

## Materials and Methods

### Data collection

The gene expression, DNA methylation along with the clinical datasets of TCGA GBMLGG was downloaded from https://tcga.xenahubs.net website. The CGGA datasets were downloaded from http://www.cgga.org.cn/index.jsp website. The glioma GEO datasets were downloaded from www.ncbi.nlm.nih.gov/geo website, including GSE4412 19, GSE43378 20, GSE13041 21, GSE44971 22, GSE68848 23, GSE74187 24, GSE83300 25 and GSE16011 26 datasets. In TCGA, CGGA, GSE4412 and GSE43378 datasets, glioma patients with transcriptional data and clinical overall survival data were selected for further studies.

### Identification of the differentially expressed genes between GBM and LGG

The differentially expressed genes between LGG and GBM patients were determined using two tails paired Student's t test in TCGA, CGGA, GSE4412 and GSE43378 datasets. Genes with fold change >2 and P value<0.001 were selected for further studies.

### Heatmap presentation

R software 'pheatmap' package (version 1.0.12, https://cran.r-project.org/web/packages/pheatmap/) was used to create the figures of heatmaps. The clustering scale and clustering distance were determined by 'average' method and 'correlation' method, respectively.

### ROC curves

R software 'pROC' package (version 1.16.2, https://cran.r-project.org/web/packages/pROC/) was used to determine the area under the ROC curve (AUC).

### Survival analysis

The survival analysis was carried out using 'survival' package (version 3.1-8, https://cran.r-project.org/web/packages/survival/index.html) in R statistics software. Kaplan-Meier estimator was used to determine the overall survival of different groups of glioma patients. P values were determined using Log-rank test. P value less than 0.05 was chosen to be significantly different. R software 'survival' package was also used for univariate cox and multivariate cox regression analysis.

### Correlation analysis

Correlation analysis was performed using “lm” method in R software. P values were determined by spearman correlation.

### Cell culture and cell proliferation

Glioma cancer cell lines A172 and U87 were cultured in MEM medium (invitrogen) supplemented with 10% FBS at 37 °C in a humidified atmosphere with 5% CO_2_. The cell proliferation was detected by CCK-8 assay. Briefly, A172 and U87 cells were seeded in 96-well plate with 10 µL CCK-8 per well. The cell growth rate (OD_450_) was measured.

### CDHR1 over-expression

CDHR1 cDNA was cloned into pCDNA 3.0 vector, and then transfected into A172 and U87 cells through lipofectamine 2000 (Invitrogen, Carlsbad, CA, USA) according to manufacturer's protocol. The over-expression of CDHR1 was validated using western blot. Anti-human β-actin and CDHR1 antibodies were purchased from Santa Cruz Biotechnology.

### Trans-well invasion assay

Trans-well invasion assays were performed in 24-well trans-well chamber with diluted matrigel (BD Biosciences, San Jose, CA, USA). The migrated cells from three random fields were photographed and counted. P values were determined using two tails paired Student's t test. P value less than 0.05 was chosen to be significantly different.

## Results

### Identification of the differentially expressed genes between GBM and LGG subtypes

**Figure 1:**
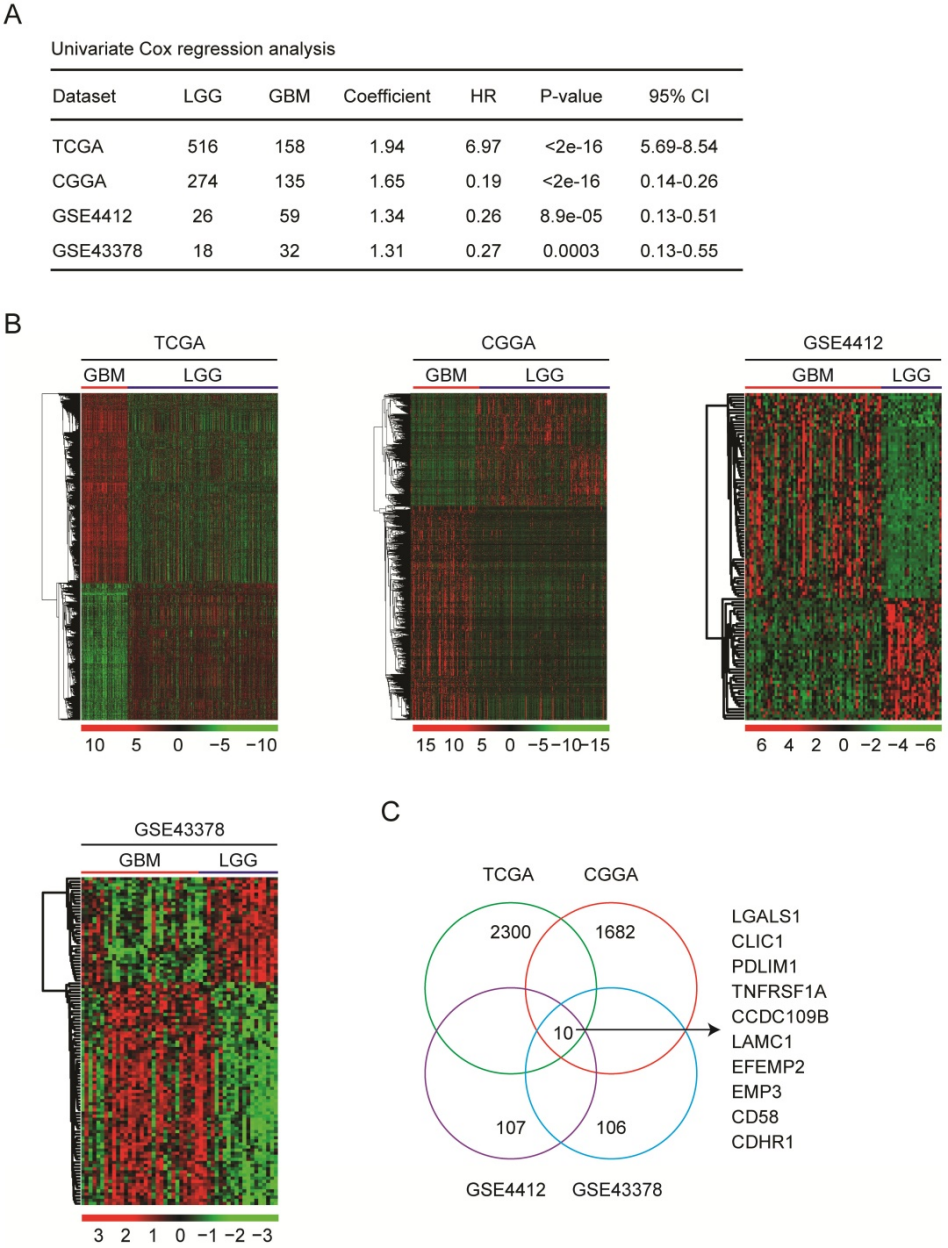
** Identification of the differentially expressed genes between GBM and LGG subtypes. (A)** The number of GBM and LGG patients in TCGA, CGGA, GSE4412 and GSE43378 datasets was demonstrated. Univariate cox regression was used to show the different clinical outcomes of GBM and LGG patients. The overall survival P-value was determined by log-rank test. HR represented hazard ratio. **(B)** Un-supervised clustering heatmaps demonstrated the differentially expressed genes (fold change>2 and P value<0.001) between GBM and LGG subtypes in TCGA, CGGA, GSE4412 and GSE43378 datasets. Up-regulated (red) and down-regulated (green) genes in GBM were delineated. **(C)** Venn diagram showed the number of differentially expressed genes between GBM and LGG in TCGA, CGGA, GSE4412 and GSE43378 datasets.

Glioma GBM subtype have worse prognosis than LGG subtype. Identification of the differentially expressed genes between GBM and LGG may provide new prognostic makers for glioma. To do that, GBM and LGG expression profiles from same dataset were studies. Totally, we collected 834 LGG and 384 GBM expression samples from TCGA 13, 14, CGGA 15, 16, GSE4412 19 and GSE43378 20 datasets. The detailed clinic parameters of glioma patients in TCGA, CGGA, GSE4412 and GSE43378 datasets were described in supplementary data. Univariate cox regression showed that GBM patients indeed had significantly shorter overall survival than LGG patients in all those four datasets (Fig. 1A).

Next, the differentially expressed genes between GBM and LGG subtypes in TCGA, CGGA, GSE4412 and GSE43378 datasets were determined. Based on the criteria of fold change>2 and P value<0.001, 2300 genes were differentially expressed in GBM patients in TCGA dataset, 1682 genes in CGGA dataset, 107 genes in GSE4412 dataset, and 106 genes in GSE43378 dataset, respectively (Fig. 1B). Venn diagram showed that ten genes CCDC109B, CD58, CDHR1, CLIC1, EFEMP2, EMP3, LAMC1, LGALS1, PDLIM1 and TNFRSF1A were commonly differentially expressed between GBM and LGG subtypes in TCGA, CGGA, GSE4412 and GSE43378 datasets (Fig. 1C).

### Compared with LGG, CDHR1 is down-regulated in GBM patients

**Figure 2:**
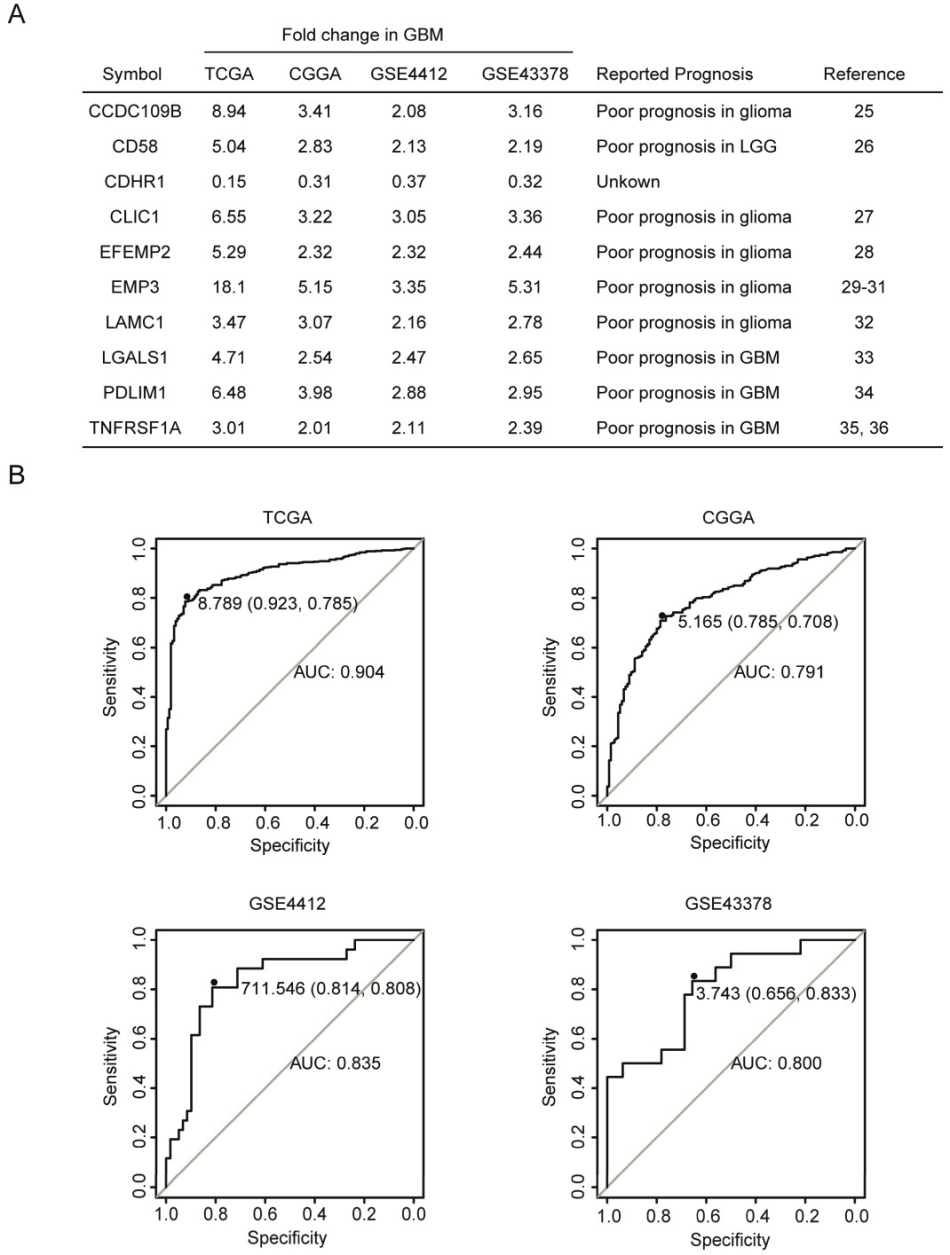
** Compared with LGG, CDHR1 is down-regulated in GBM patients. (A)** Compared with LGG, the fold changes of the ten commonly differentially expressed genes in GBM were demonstrated in TCGA, CGGA, GSE4412 and GSE43378 datasets. The previously reported prognostic effects of CCDC109B, CD58, CLIC1, EFEMP2, EMP3, LAMC1, LGALS1, PDLIM1 and TNFRSF1A in glioma were also demonstrated by literature research.** (B)** ROC curves showed the specificity and sensitivity of using expression levels of CHDR1 to distinguish GBM from LGG in TCGA, CGGA, GSE4412 and GSE43378 datasets.

The fold changes of CCDC109B, CD58, CDHR1, CLIC1, EFEMP2, EMP3, LAMC1, LGALS1, PDLIM1 and TNFRSF1A in GBM were further illustrated in Fig. 2A. Compared with LGG, CCDC109B, CD58, CLIC1, EFEMP2, EMP3, LAMC1, LGALS1, PDLIM1 and TNFRSF1A were all highly expressed in GBM. Particularly, EMP3 was most significantly up-regulated in GBM patients. Compared with LGG, there were 18.1, 5.15, 3.35 and 5.31 fold changes of EMP3 in GBM patients in TCGA, CGGA, GSE4412 and GSE43378, respectively (Fig. 2A). On the contrary, CDHR1 was the only gene which was down-regulated in GBM patients. Compared with LGG, there were 0.15, 0.31, 0.37 and 0.32 fold changes of CDHR1 in GBM patients in TCGA, CGGA, GSE4412 and GSE43378, respectively (Fig. 2A). Moreover, ROC analysis in TCGA, CGGA, GSE4412 and GSE43378 datasets showed that expression levels of CDHR1 could distinguish GBM from LGG patients with high specificity and sensitivity (Fig. 2B).

Consistent with the high expression in GBM subtype, previous reports suggested that CCDC109B 27, CD58 28, CLIC1 29, EFEMP2 30, EMP3 31-33, LAMC1 34, LGALS1 35, PDLIM1 36 and TNFRSF1A 37, 38 were all bad prognostic factors in glioma, as were summarized in Fig. 2A. However, the prognostic effects of CDHR1 in glioma were never reported and searching with the keywords of “CDHR1 in glioma” had failed to retrieve any related publication in PubMed. We speculated that, like other nine genes, CDHR1 was a potential prognostic marker in glioma. So, the expression and prognostic effects of CDHR1 in glioma were further studied.

### CDHR1 is lowly expressed in glioma tissues and low expression of CDHR1 is a bad prognostic factor of glioma

**Figure 3:**
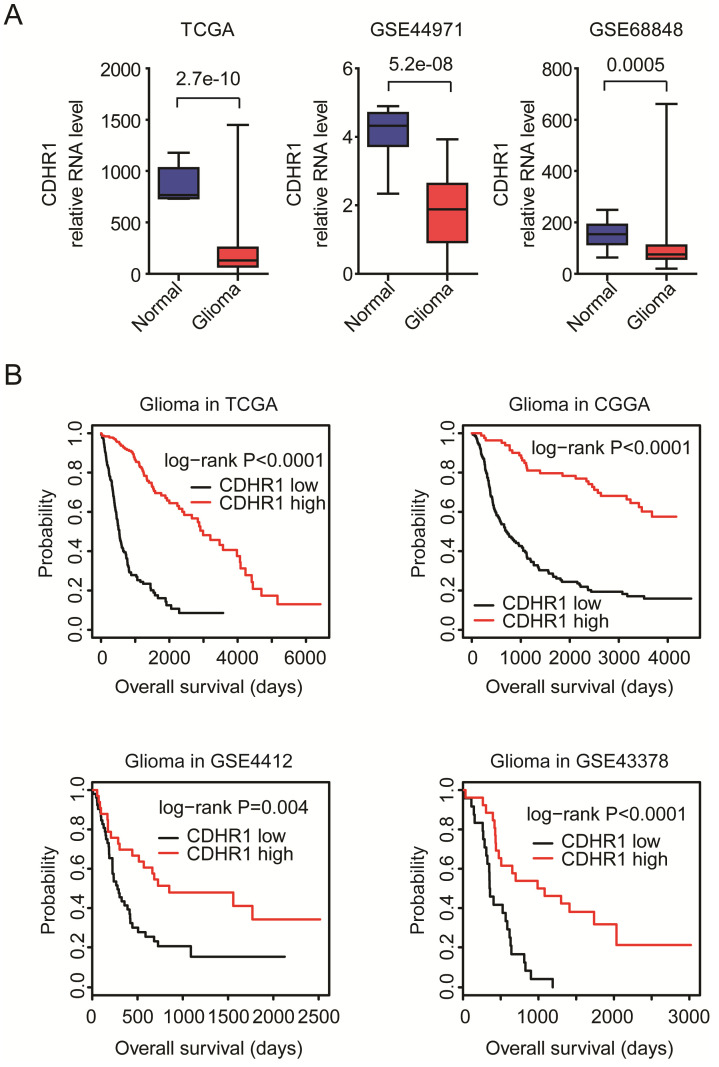
** CDHR1 is lowly expressed in glioma and low expression of CDHR1 is a bad prognostic factor of glioma. (A)** Box plots demonstrated the different expression levels of CDHR1 in normal brain tissues (blue) and glioma tissues (red). P values were generated by using the two tails paired Student's t test. **(B)** The Kaplan-Meier Plotters demonstrated the associations between expression levels of CDHR1 and glioma overall survival using TCGA, CGGA, GSE4412 and GSE43378 datasets. The P-values showed the different overall survival of CDHR1 highly expressed glioma (red) and CDHR1 lowly expressed glioma patients (black).

First, the expression levels of CDHR1 in normal brain tissues and glioma tissues were tested. Totally, 42 normal brain tissues and 446 glioma tissues were collected from TCGA 13, 14, GSE44971 22 and GSE68848 23 datasets. Compared with normal brain tissues, the expression levels of CDHR1 were significantly down-regulated in glioma tissues in all TCGA, GSE44971 and GSE68848 datasets (Fig. 3A).

Second, the associations between expression levels of CDHR1 and glioma overall survival were tested using TCGA, CGGA, GSE4412 and GSE43378 datasets. We found that, consistent with the down-regulation of CDHR1 in GBM, the low expression of CDHR1 was a bad prognosis in glioma. Glioma patients with higher expression of CDHR1 had longer overall survival than glioma patients with lower expression of CDHR1 in all TCGA, CGGA, GSE4412 and GSE43378 datasets (Fig. 3B).

### CDHR1 is lowly expressed in mesenchymal GBM subtype and low expression of CDHR1 is a bad prognostic factor of GBM

**Figure 4:**
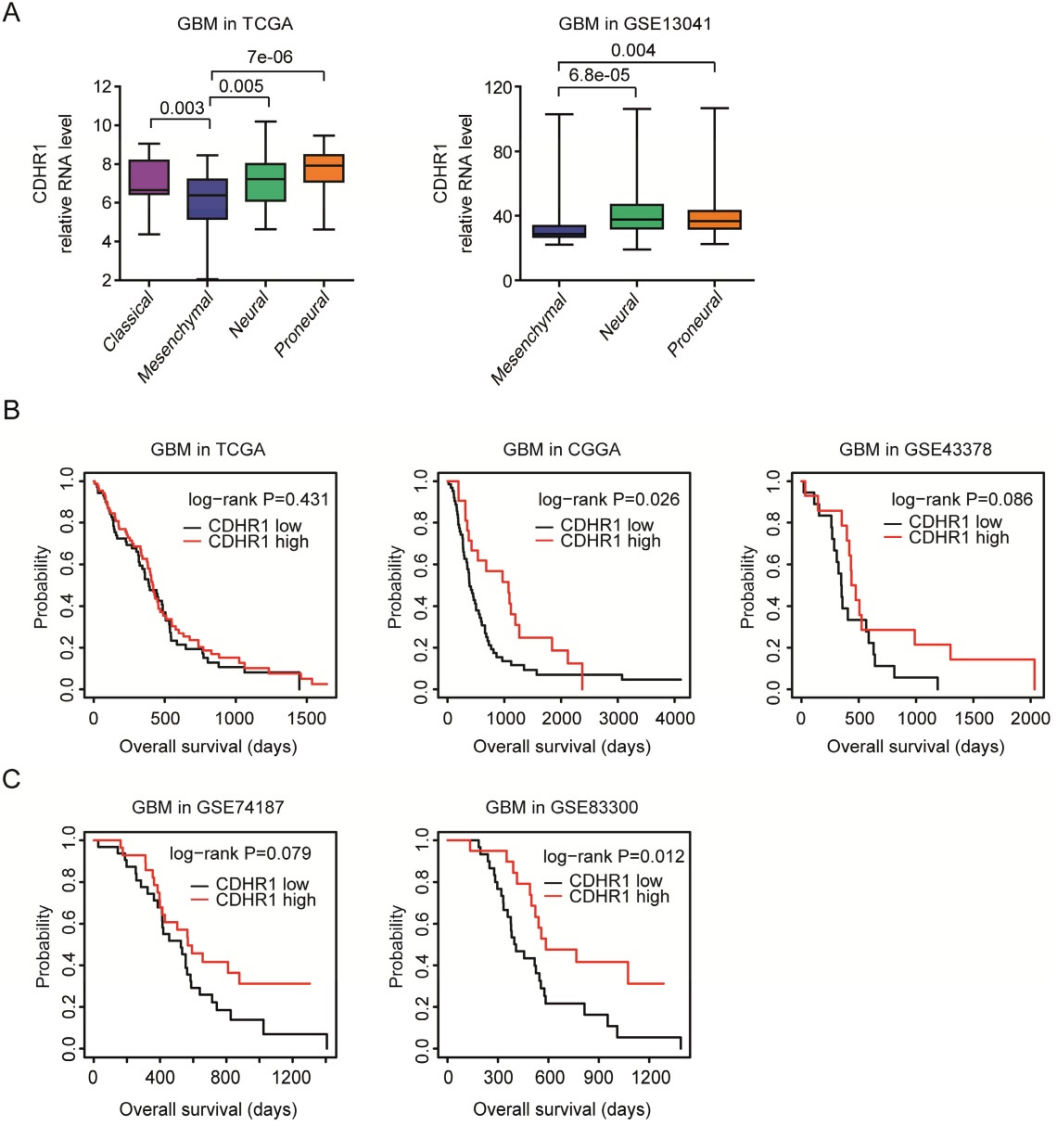
** CDHR1 is lowly expressed in mesenchymal GBM subtype and low expression of CDHR1 is a bad prognostic factor of GBM. (A)** Box plots demonstrated the different expression levels of CDHR1 in classical, mesenchymal, neural and proneural GBM subtypes in TCGA and GSE13041 datasets. **(B)** The Kaplan-Meier Plotters demonstrated the different overall survival of CDHR1 highly expressed GBM (red) with CDHR1 lowly expressed GBM patients (black) in TCGA, CGGA and GSE43378 datasets. **(C)** The Kaplan-Meier Plotters demonstrated the prognostic effects of CDHR1 in GBM patients derived from GSE74187 and GSE83300 datasets.

As a subtype of glioma, GBM could be further divided into classical, mesenchymal, neural and proneural four subtypes based on the molecular alterations and mesenchymal subtype of GBM had unfavorable prognosis 39. We found that, compared with classical, neural and proneural GBM subtypes, CDHR1 was down-regulated in mesenchymal subtype of GBM in TCGA dataset (Fig. 4A). The low expression levels of CDHR1 in mesenchymal subtype of GBM were further validated in GSE13041 dataset 21 (Fig. 4A).

The prognostic effects of CDHR1 were further tested in glioma GBM subtype. As showed in the Kaplan-Meier Plotters, there was no significantly different overall survival in CDHR1 highly expressed GBM or CDHR1 lowly expressed GBM patients in TCGA and GSE43378 datasets (Fig. 4B). However, in CGGA dataset, CDHR1 highly expressed GBM patients had significantly longer overall survival than CDHR1 lowly expressed GBM patients (Fig. 4B). The prognostic effects of CDHR1 in GBM were further tested using GSE74187 24 and GSE83300 25 datasets. Similar to the results derived from CGGA dataset, low expression of CDHR1 was a significantly bad prognosis of GBM in GSE83300 dataset, but not significant in GSE74184 dataset (Fig. 4C).

### CDHR1 is lowly expressed in astrocytoma LGG subtype and low expression of CDHR1 is a bad prognostic factor of LGG

**Figure 5:**
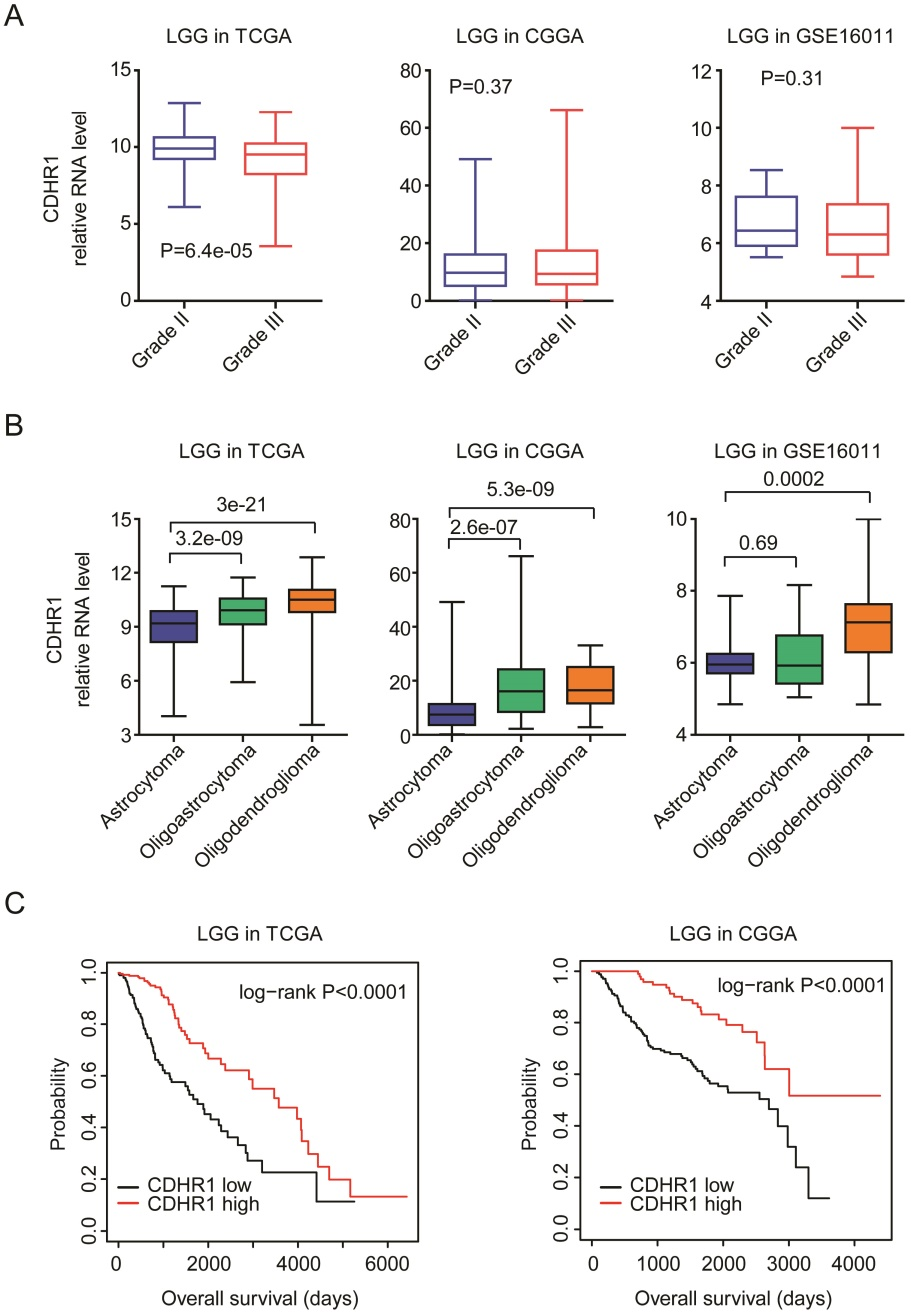
** CDHR1 is lowly expressed in astrocytoma LGG subtype and low expression of CDHR1 is a bad prognostic factor of LGG. (A)** Box plots demonstrated the different expression levels of CDHR1 in grade II and grade III LGG subtypes in TCGA, CGGA and GSE16011 datasets. **(B)** Box plots demonstrated the different expression levels of CDHR1 in astrocytoma, oligoastricytoma and oligodendroglioma LGG subtypes in TCGA, CGGA and GSE16011 datasets. **(C)** The Kaplan-Meier Plotters demonstrated the different overall survival of CDHR1 highly expressed LGG (red) with CDHR1 lowly expressed LGG patients (black) in TCGA and CGGA datasets.

LGG is grade II-III glioma. We further showed that, compared with grade II LGG, CDHR1 was down-regulated in grade III LGG patients in TCGA dataset (Fig. 5A). However, in CGGA and GSE16011 26 datasets, there was no significant difference of CDHR1 expression between grade II or III LGG patients (Fig. 5A). LGG could be further divided into astrocytoma, oligoastricytoma and oligodendroglioma subtypes 7. Next, we evaluated the expression levels of CDHR1 in different LGG subtypes. In TCGA and CGGA datasets, we showed that, compared with oligoastricytoma and oligodendroglioma, CDHR1 was down-regulated in astrocytoma subtype of LGG (Fig. 5B). Moreover, in GSE16011 dataset, the expression of CDHR1 was also lower in astrocytoma LGG subtype, compared with oligodendroglioma LGG subtype (Fig. 5B).

The prognostic effects of CDHR1 were also tested in glioma LGG subtype. CDHR1 highly expressed LGG patients had significantly longer overalls survival than CDHR1 lowly expressed LGG patients in TCGA and CGGA datasets (Fig. 5C). Those results suggested that CDHR1 was also a prognostic factor in glioma GBM or LGG subtypes, respectively.

### CDHR1 is highly expressed in IDH mutant glioma and high expression of CDHR1 is a favorable prognosis in IDH mutant or IDH wild type LGG patients

**Figure 6:**
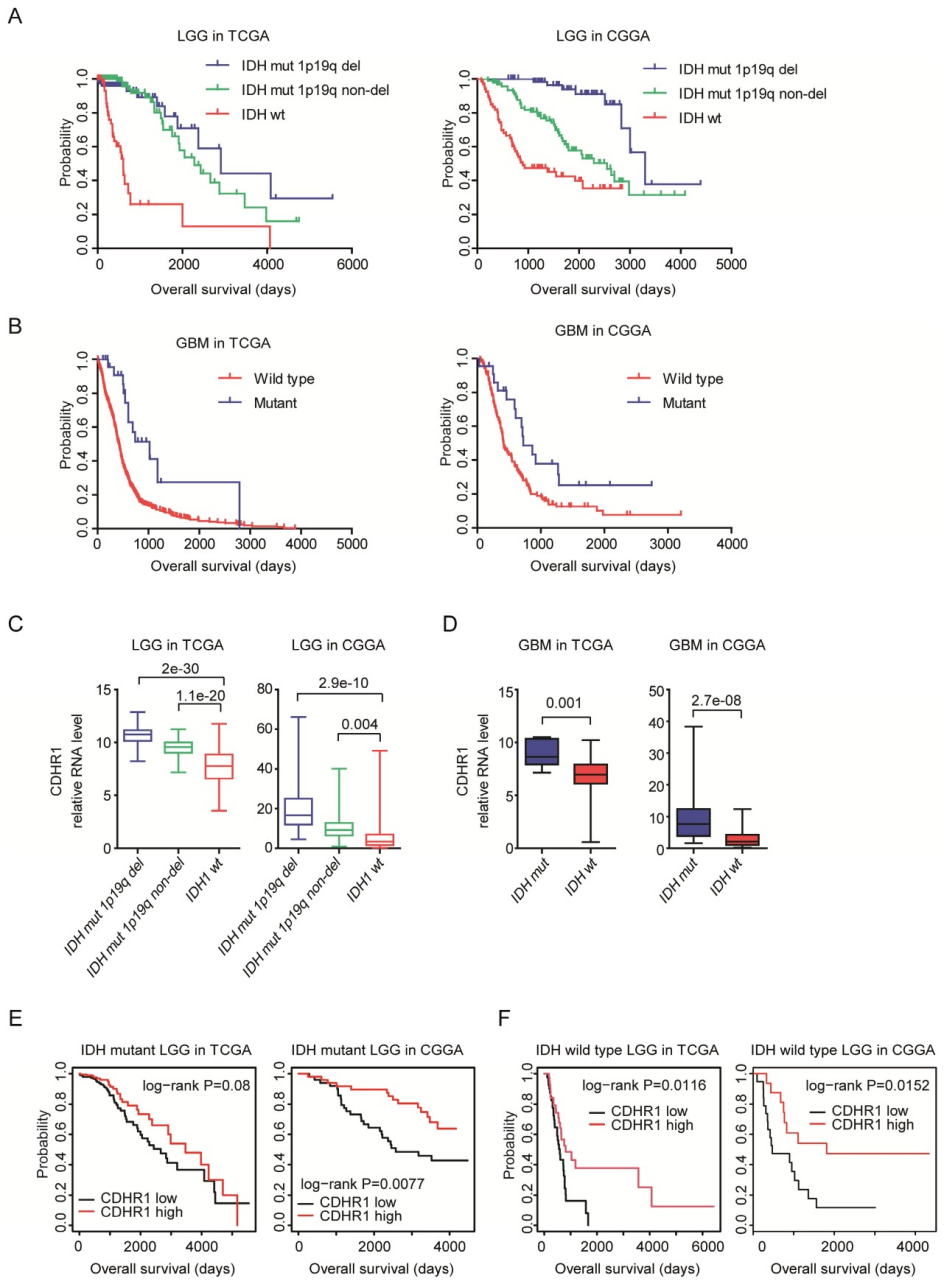
** CDHR1 is highly expressed in IDH mutant glioma and high expression of CDHR1 is a favorable prognosis in IDH mutant or IDH wild type LGG patients. (A)** The Kaplan-Meier Plotters demonstrated the different clinical outcomes of LGG patients with IDH mutation 1p19q deletion, IDH mutation 1p19q non-deletion or without IDH mutation. **(B)** The Kaplan-Meier Plotters demonstrated the different clinical outcomes of IDH mutant and IDH wild type GBM patients. **(C)** Box plots demonstrated the different expression levels of CDHR1 in IDH mutation 1p19q deletion, IDH mutation 1p19q non-deletion and IDH wild type subtypes of LGG in TCGA and CGGA datasets. **(D)** Box plots demonstrated the different expression levels of CDHR1 in IDH mutant and IDH wild type subtypes of GBM in TCGA and CGGA datasets. **(E)** The Kaplan-Meier Plotters demonstrated the prognostic effects of CDHR1 in IDH mutant LGG patients derived from TCGA and CGGA datasets. **(F)** The Kaplan-Meier Plotters demonstrated the prognostic effects of CDHR1 in IDH wild type LGG patients derived from TCGA and CGGA datasets.

IDH mutation is a common mutation in glioma. IDH mutant glioma patients have better clinical outcomes 40, 41. Indeed, in both TCGA and CGGA datasets, IDH wild type LGG patients had significantly shorter overall survival than LGG patients with IDH mutations (Fig. 6A). Similarly, in GBM patients, IDH mutation was associated with better clinical outcomes in TCGA and CGGA datasets (Fig. 6B).

We then studied the different expression levels of CDHR1 in glioma patients with or without IDH mutations. First, CHDR1 expression was lower in IDH wild type LGG patients, compared with LGG patients with IDH mutation 1p19q deletion or IDH mutation 1p19q non-deletion in both TCGA and CGGA datasets (Fig. 6C). IDH was also highly expressed in GBM patients with IDH mutation, compared with IDH wild type GBM patients in TCGA and CGGA datasets (Fig. 6D). Moreover, CDHR1 low expression was associated with the unfavorable prognosis of IDH mutant LGG patients in CGGA datasets (Fig. 6E). However, in TCGA datasets, there was no significantly different overall survival between CHDR1 highly or lowly expressed IDH mutant LGG patients (Fig. 6E). Furthermore, in IDH wild type LGG patients, CDHR1 low expression was associated with the unfavorable prognosis in TCGA and CGGA datasets (Fig. 6F).

### CDHR1 expression is correlated with MGMT methylation intensity

**Figure 7:**
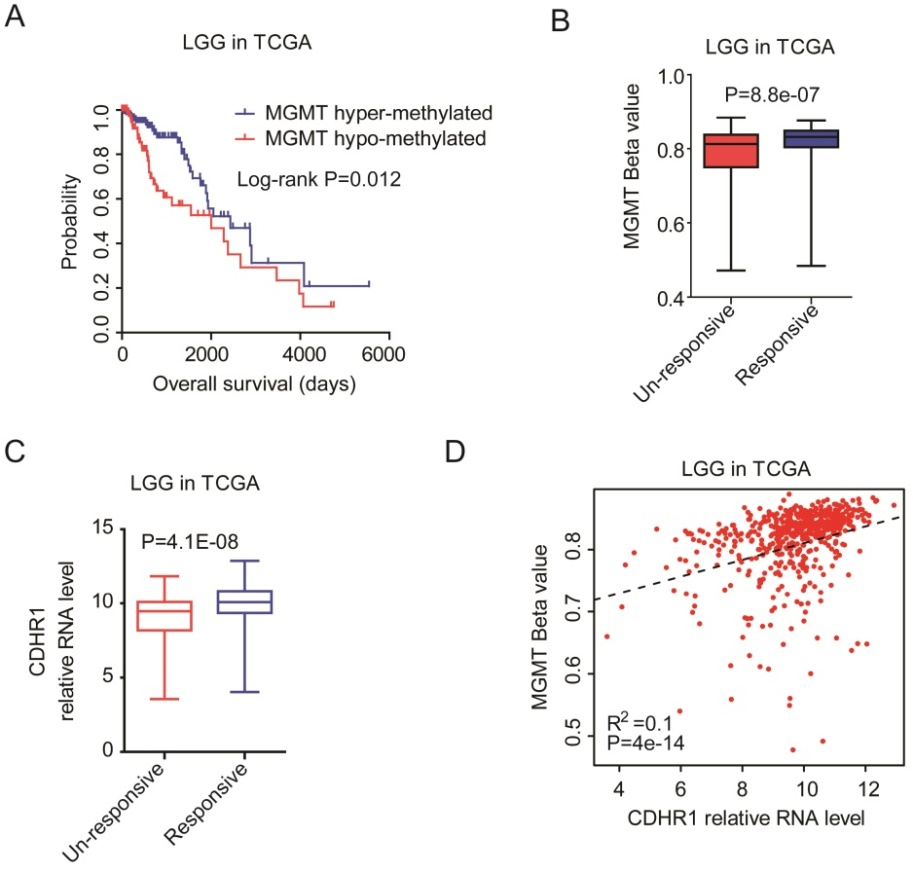
** CDHR1 expression is correlated with MGMT methylation intensity. (A)** The Kaplan-Meier Plotters demonstrated the different clinical outcomes of LGG patients with MGMT hypo-methylation or with MGMT hyper-methylation. **(B)** Box plots demonstrated the different methylation intensity of MGMT in chemotherapy responsive or chemotherapy un-responsive LGG patients. **(C)** Box plots demonstrated the different expression levels of CDHR1 in chemotherapy responsive or chemotherapy un-responsive LGG patients. **(D)** Spearman correlation between CDHR1 expression and MGMT methylation in LGG patients in TCGA dataset.

MGMT methylation was another important prognostic factor in glioma. Epigenetic hyper-methylation of MGMT could increase the sensitivity of temozolomide therapy, and represented a favorable prognostic maker 42-44. We showed that in TCGA dataset, LGG patients with hyper-methylated MGMT had longer overall survival than LGG patients with hypo-methylated MGMT (Fig. 7A). Moreover, the methylation levels of MGMT were higher in chemotherapy responsive LGG patients than chemotherapy un-responsive LGG patients (Fig. 7B).

On the contrary, the expression levels of CDHR1 were higher in chemotherapy responsive LGG patients than chemotherapy un-responsive LGG patients (Fig. 7C). Also, we observed significantly positive correlations of CDHR1 expression and MGMT methylation (Fig. 7D). All those results suggested that CDHR1 was associated with IDH mutation and MGMT methylation, and CDHR1 was an important prognostic factor of glioma.

### CDHR1 is an independent prognostic factor and combination of CDHR1 with EMP3 achieves better clinical predication

**Figure 8:**
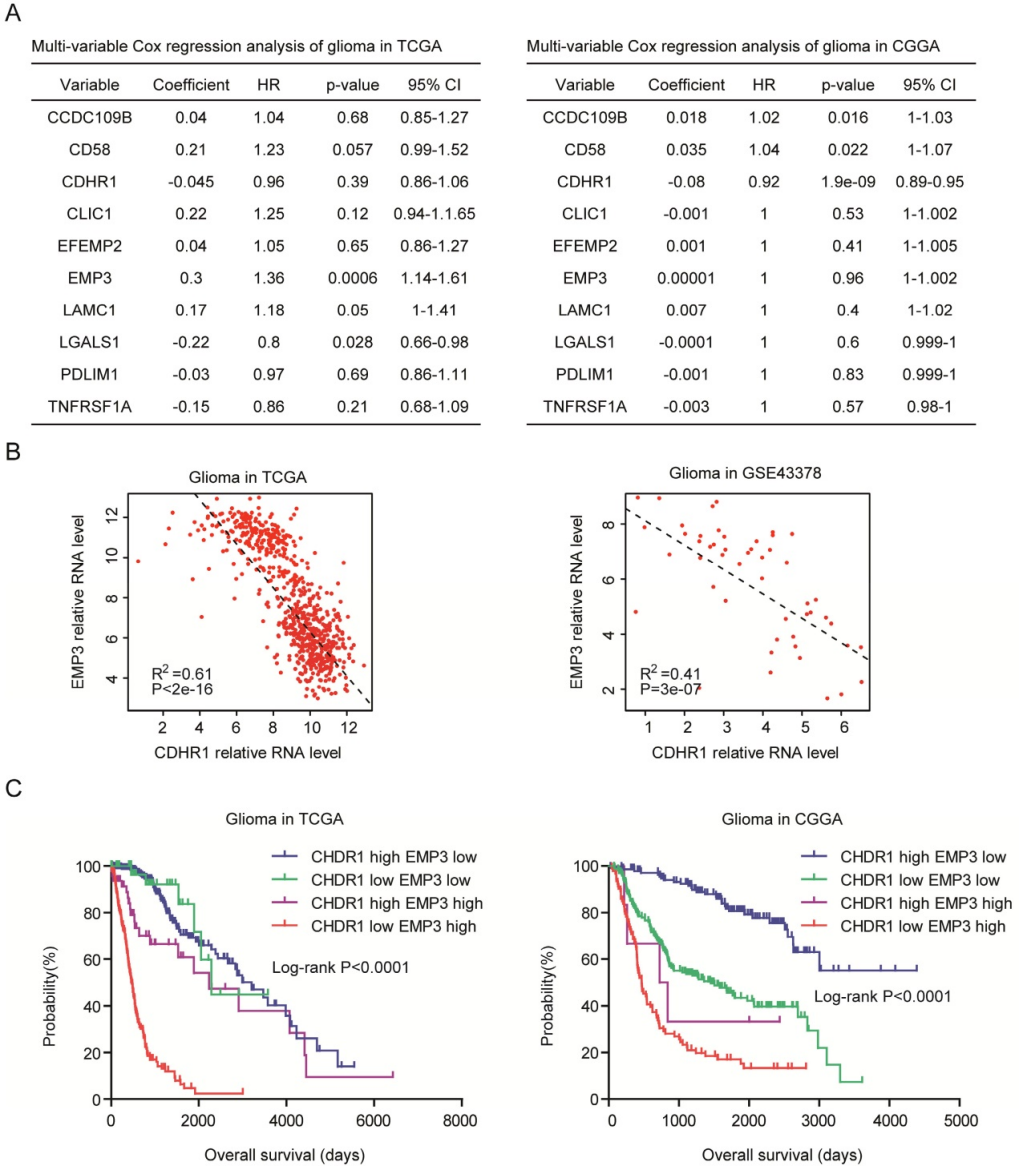
** CDHR1 is an independent prognostic factor and combination of CDHR1 with EMP3 achieves better clinical predication. (A)** Multivariate cox regression was used to test the relationships of CCDC109B, CD58, CDHR1, CLIC1, EFEMP2, EMP3, LAMC1, LGALS1, PDLIM1, TNFRSF1A expressions and overall survival in glioma patients in TCGA and CGGA datasets. **(B)** Spearman correlation between CDHR1 and EMP3 expression in glioma patients was tested in TCGA and GSE43378 datasets. **(C)** Kaplan-Meier plotters demonstrated the different overall survival of glioma patients with different expression levels of EMP3 and CDHR1 in TCGA and CGGA datasets. Log-rank test was used to determine the P values.

Next, we determined the relationships of CDHR1 with CCDC109B, CD58, CLIC1, EFEMP2, EMP3, LAMC1, LGALS1, PDLIM1 and TNFRSF1A. Multivariate cox regression showed that EMP3 and LGALS1 were independent prognostic factors in TCGA dataset (Fig. 8A), and in CGGA dataset, CDHR1 was an independent prognostic factor (Fig. 8A). Previously, our results showed that EMP3 was highly expressed in old LGG patients and was a critical prognostic factor in glioma 33. We observed significantly negative correlations between CDHR1 expression and EMP3 expression in both TCGA and GSE43378 datasets (Fig. 8B).

Furthermore, we determined the synergistic prognosis of CDHR1 with EMP3. We found that glioma patients with CDHR1 low EMP3 high expression had worse clinical outcomes in TCGA and CGGA datasets (Fig. 8C). While, glioma patients with CDHR1 high EMP3 low expression had better clinical outcomes (Fig. 8C). Those results suggested that combination of CDHR1 and EMP3 may achieve better prognostic significance than CDHR1 or EMP3 alone.

### Over-expression of CDHR1 inhibits glioma cell growth and invasion

**Figure 9:**
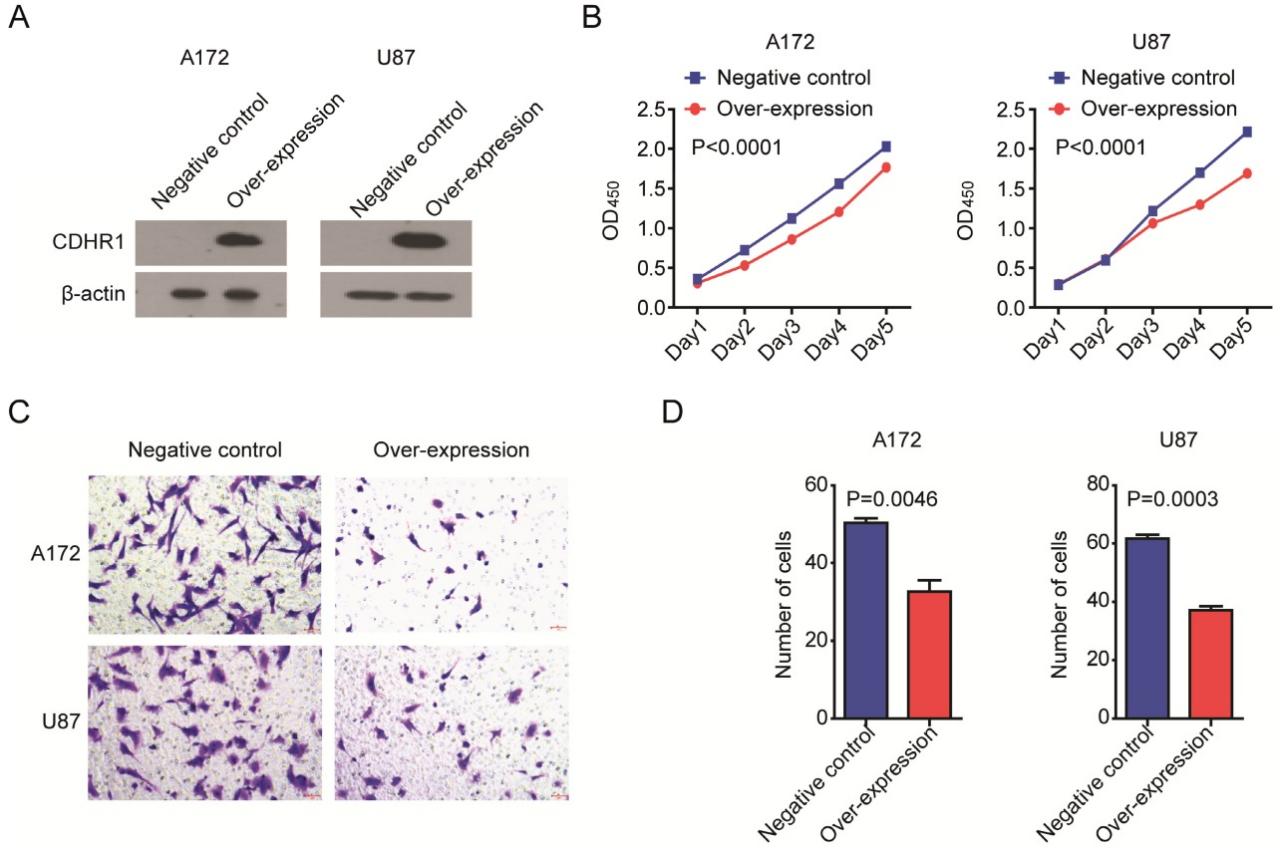
** Over-expression of CDHR1 inhibits glioma cell growth and invasion. (A)** Western blot showed the over-expression of CDHR1 in glioma A172 and U87 cell lines, using an empty vector as negative control. **(B)** Cell growth in A172 and U87 with or without CDHR1 over-expression was detected using CCK8 assay. The cell growth rate was determined by OD_450_. **(C)** Representative images of invasive A172 and U87 cells with or without CDHR1 over-expression in trans-well assay. **(D)** Statistical quantification of migrated A172 and U87 cells with or without CDHR1 over-expression in trans-well assay. Results were representative of three independent experiments.

At last, we determined the biological functions of CDHR1 in glioma. Two glioma cell lines A172 and U87 were transfected with CDHR1 over-expression plasmid. Western blot showed the high expression of CDHR1 in A172 and U87 cells after plasmid transfection (Fig. 9A). Then, we tested the cell growth using CCK8 assay. We found that, the cell growth was significantly inhibited by CDHR1 over-expression in both A172 and U87 cells (Fig. 9B). Furthermore, over-expression of CDHR1 inhibited glioma cell invasion as tested using trans-well assay (Fig. 9C). The number of invasive cells was significantly suppressed by CDHR1 over-expression in A172 and U87 cells (Fig. 9D). Those results were consistent with our previous results that CDHR1 was lowly expressed in glioma cells and low expression of CDHR1 was an unfavorable prognostic factor in glioma.

## Discussion

Glioma is a heterogeneous disease. Histological grades, IDH mutation 40, 41, MGMT methylation 42-44 and EMP3 expression 31-33 were all validated prognostic factors of glioma. Interesting, those prognostic factors were coordinated with each other. For example, EMP3 was over-expressed in grade IV glioma (GBM). And grade II-III glioma (LGG) had high frequency of IDH mutation 11, 45. In our studies, using TCGA, CGGA and GEO datasets, we further confirmed the prognostic significance of histological grades, IDH mutation, MGMT methylation and EMP3 expression. Moreover, we believed that genes associated with histological grades, IDH mutation, MGMT methylation and EMP3 expression in glioma were also potential prognostic factors for glioma.

In this analysis, we started with the differentially expressed genes between GBM and LGG. We identified ten differentially expressed genes shared in TCGA, CGGA, GSE4412 and GSE43378 datasets. Among them, nine genes, including EMP3 were previously reported prognostic makers of glioma. However, the expressions and prognostic effects of CDHR1 were never known. We showed that, CDHR1 was down-regulated in GBM and CDHR1 was down-regulated in IDH mutant glioma patients. Moreover, CDHR1 expression was positively correlated with MGMT methylation and negatively correlated with EMP3 expression. All those features suggested that CDHR1 was a potential prognostic maker.

Indeed, CDHR1 was down-regulated in glioma tissues, compared with normal brain tissues. Moreover, the down-regulation of CDHR1 was associated with poor clinical outcomes of glioma in all TCGA, CGGA, GSE4412 and GSE43378 datasets. Furthermore, the bad prognostic effects of CDHR1 were detected in GBM or LGG patients in at least one dataset. GBM or LGG patients with CDHR1 high expression had longer overall survival. At last, we confirmed the function of CDHR1 by over-expression CDHR1 in glioma cell lines. The over-expression of CDHR1 could significantly inhibit glioma cell growth and cell invasion. All those results suggested that low expression of CDHR1 was an independent unfavorable prognostic factor in glioma.

Our analysis highlighted that genes differentially expressed in different glioma grades were potential prognostic factors. Next, it is interesting to identify the differentially expressed genes between IDH mutant and IDH wild type glioma patients or identify the genes most associated with MGMT methylation and EMP3 expression. Those analyses may provide new understandings of the development of glioma and suggest novel therapeutic targets and prognostic makers for glioma.

## Conclusion

Low expression of CDHR1 was an independent unfavorable prognostic factor in glioma.

## Supplementary Material

Supplementary data.

## Abbreviations

LGG: Lower grade glioma
GBM: glioblastoma
TCGA: The Cancer Genome Atlas
CGGA: Chinese Glioma Genome Atlas
CDHR1: Cadherin-related family member 1
GEO: Gene Expression Omnibus
HR: hazard ratio
